# Biosynthesis of Silver Nanoparticles Using *Taxus* *yunnanensis* Callus and Their Antibacterial Activity and Cytotoxicity in Human Cancer Cells

**DOI:** 10.3390/nano6090160

**Published:** 2016-09-01

**Authors:** Qian Hua Xia, Yan Jun Ma, Jian Wen Wang

**Affiliations:** College of Pharmaceutical Sciences, Soochow University, Suzhou 215123, China; qianhuaxia@aliyun.com (Q.H.X.); yjunma@aliyun.com (Y.J.M.)

**Keywords:** *Taxus yunnanensis*, callus extract, silver nanoparticles, antibacterial, cytotoxicity

## Abstract

Plant constituents could act as chelating/reducing or capping agents for synthesis of silver nanoparticles (AgNPs). The green synthesis of AgNPs has been considered as an environmental friendly and cost-effective alternative to other fabrication methods. The present work described the biosynthesis of AgNPs using callus extracts from *Taxus yunnanensis* and evaluated their antibacterial activities in vitro and potential cytotoxicity in cancer cells. Callus extracts were able to reduce silver nitrate at 1 mM in 10 min. Transmission electron microscope (TEM) indicated the synthesized AgNPs were spherical with the size range from 6.4 to 27.2 nm. X-ray diffraction (XRD) confirmed the AgNPs were in the form of nanocrystals. Fourier transform infrared spectroscopy (FTIR) suggested phytochemicals in callus extracts were possible reducing and capping agents. The AgNPs exhibited effective inhibitory activity against all tested human pathogen bacteria and the inhibition against Gram-positive bacteria was stronger than that of Gram-negative bacteria. Furthermore, they exhibited stronger cytotoxic activity against human hepatoma SMMC-7721 cells and induced noticeable apoptosis in SMMC-7721 cells, but showed lower cytotoxic against normal human liver cells (HL-7702). Our results suggested that biosynthesized AgNPs could be an alternative measure in the field of antibacterial and anticancer therapeutics.

## 1. Introduction

As an important antibacterial agent, silver has been used for many years [1]. Recently, silver nanoparticles (AgNPs) promoted the usage of silver in biomedical applications with its unique physic-chemical, optical and biological properties [2,3]. Several methods have been developed for the synthesis of AgNPs, including physical, chemical and biological methods [4,5]. The biological synthetic methods are preferred because they are safe, cheap and environment-friendly, whereas other physical and chemical methods require energy, high pressure, temperature and chemicals toxic to biological systems. Plants and microbes are currently used for AgNP synthesis among the biological synthetic methods [6].

Recently, a novel use of plant callus to synthesize AgNPs has been widely investigated. Many kinds of plant callus induced from papaya, alfalfa, *Catharanthus* *roseus* and *Sesuvium* *portulacastrum* have been applied in the green synthesis of AgNPs [7,8,9,10]. Applying plant callus in synthesizing AgNPs avoids various drawbacks including low yield, high expense, energy consumption in the physical approaches, and contamination in the wet-chemical procedure [11]. As an incessant source in laboratory, callus culture overcomes the difficulties of scarcity in wild plant source [8]. Interestingly, callus extracts were more efficient in the fabrication of AgNPs and the synthesized AgNPs were more distinct and scattered in distribution than those synthesized by intact leaf extract [10]. The AgNPs biologically synthesized by callus showed excellent antibacterial effect against human pathogenic microorganisms [9,10]. AgNPs synthesized by calli of *Citrullus* *colocynthis* were reported to have cytotoxic activity against human epidermoid larynx carcinoma cells [12].

*Taxus* *yunnanensis* Cheng et L.K. Fu (Taxaceae), an evergreen tree endemic to Yunnan of China, is a promising source of potent anticancer paclitaxel and taxane-type diterpenes [13]. Due to the enormous commercial value of paclitaxel and the scarcity of *T. yunnanensis* trees in China, callus and cell cultures have been established as a practical alternative source for paclitaxel production [14]. Many studies on cell culture of *T. yunnanensis* focused on enhancing paclitaxel production by means of elicitation, ultrasound treatment and medium renewal [15,16]. However, little has been reported on biosynthesized AgNP using *Taxus* tree or callus. Therefore, as a follow-up to our efforts on exploring AgNPs for controlling microbes [17] and biotechnological application of *T. yunnanensis* [16], we therefore wish to report a biological method to synthesize AgNPs using the callus extracts from *T. yunnanensis*. The antibacterial and cytotoxic activity of the biosynthesized AgNPs was also investigated.

## 2. Results

### 2.1. The Synthesis of AgNPs

**Figure 1:**
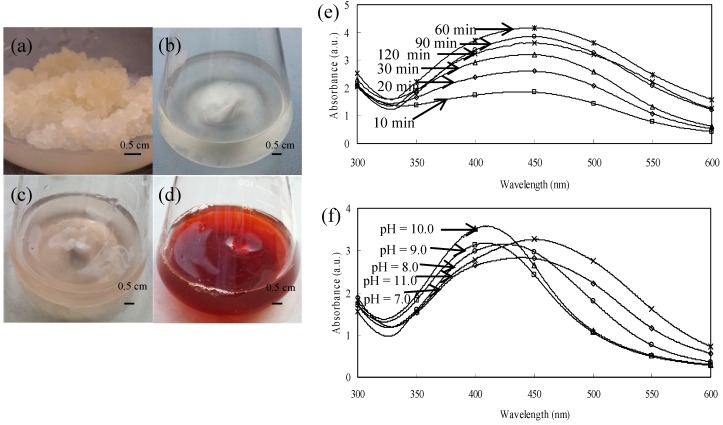
Callus of *T. yunnanensis* (**a**); and color intensity of the extracts incubated with solution of silver ions at the beginning (**b**); after 10 min (**c**); and after 120 min of reaction (**d**). UV-visible absorbance of AgNPs synthesized with the extracts under different reaction times (**e**) and different pH (**f**).

Color change of callus extracts incubated with 1 mM AgNO_3_ was observed, as shown in Figure 1a–d. The reaction solution showed a UV-visible absorbance at 449 nm because of the surface plasmon resonance of AgNPs [18] and the absorbance increased with the reaction time but decreased after 60 min of the reaction (Figure 1e). AgNPs with pH of 7, 8, 9, 10 and 11 showed maximum absorbance at 439, 425, 411, 409 and 450 nm, respectively. There was a blue shift of absorbance from pH 7 to 10, indicating the formation of smaller size nanoparticles [19]. Therefore we chose pH 10 as the optimal condition.

### 2.2. Characterization of AgNPs

**Figure 2:**
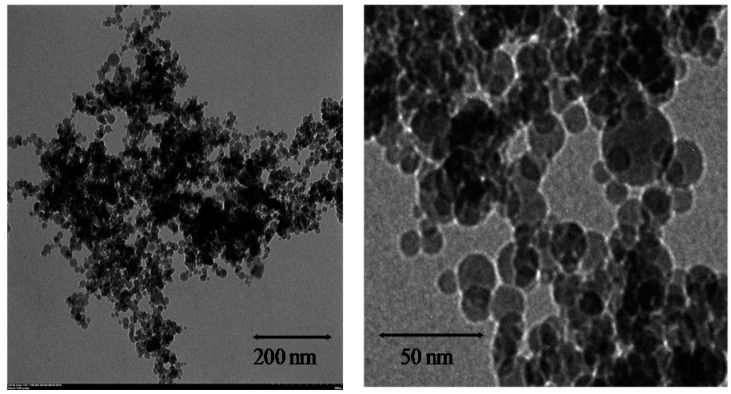
Transmission electron microscope (TEM) analysis of AgNPs synthesized with callus extracts from *T. yunnanensis* and AgNO_3_.

**Figure 3:**
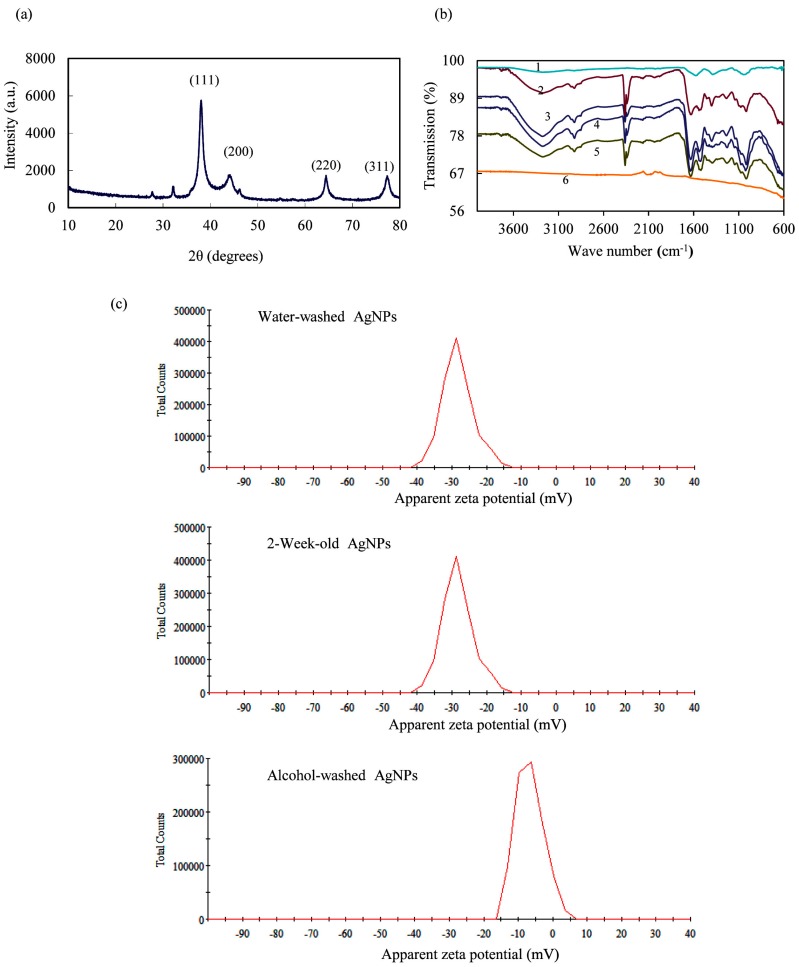
(**a**) X-ray diffraction (XRD) pattern; (**b**) Fourier transform infrared spectroscopy (FTIR) spectra (1, callus extract; 2, NB medium-washed AgNPs; 3, RPMI 1640 medium-washed AgNPs; 4, synthesized AgNPs; 5, water-washed AgNPs; 6, alcohol-washed AgNPs); and (**c**) zeta potential of AgNPs.

The biosynthesized AgNPs were characterized using transmission electron microscope (TEM) (Figure 2), X-ray diffraction (XRD) and Fourier transform infrared spectroscopy (FTIR) (Figure 3). The nanoparticles were in spherical shape. The particle size of the synthesized AgNPs shown by TEM ranged from 6.4 to 27.2 nm measured by Image J software.

The XRD of AgNPs (Figure 3a) exhibited intense peaks in the whole spectrum of 2θ value ranging from 10 to 70. Diffraction peaks at 2θ values of 38.06°, 44.11°, 64.43° and 77.33° were corresponded to the (111), (200), (220) and (311) crystal faces of face-centered silver, respectively, which was in accordance with the values in standard silver card (JCPDS No. 04-0783). Figure 3b showed the FTIR spectra of aqueous callus extracts, synthesized AgNPs as well as AgNPs washed with different solution. FTIR spectra of AgNPs showed the main peaks at 1625 and 1518 cm^−1^, which could be assigned to frame vibration of aromatic ring. Peaks at 2924 cm^−1^ corresponded to asymmetric stretching of C–H bonds. The bands at 2162 and 2033 cm^−1^ were assigned to CO adsorbed and the band round 1979 cm^−1^ was due to the overtone of C–H out of plane bending. Peaks at 1541, 1456 and 1400 cm^−1^ corresponded to amide II, methylene scissoring vibration and methyl group of proteins, respectively. Another band at 800–600 cm^−1^ could be attributed to C-H out of plane bend of aromatic phenol. Besides, the FTIR spectrum of synthesized AgNPs was similar with that of water-washed and medium-washed AgNPs, indicating most groups attached to AgNPs could not be removed by washing with water or medium. Thus, groups around AgNPs were stable and would not release into the medium in both antibacterial and cytotoxicity experiments. Furthermore, no band was observed when AgNPs was washed with alcohol. This result indicated most groups around AgNPs could be removed by alcohol. The surface zeta potentials of water-washed AgNPs, two-week-old AgNPs and alcohol-washed AgNPs were −28.8, −28.3 and −6.64 mV, respectively (Figure 3c). That is, our AgNPs was stable in the water even after two weeks but the stability decreased after washed by alcohol. Besides, the visible precipitates could be seen and after alcohol washing the dry powder of AgNPs could not be dispersed in water (Figure S1), indicating the colloidal stability of AgNPs was broken.

**Figure 4:**
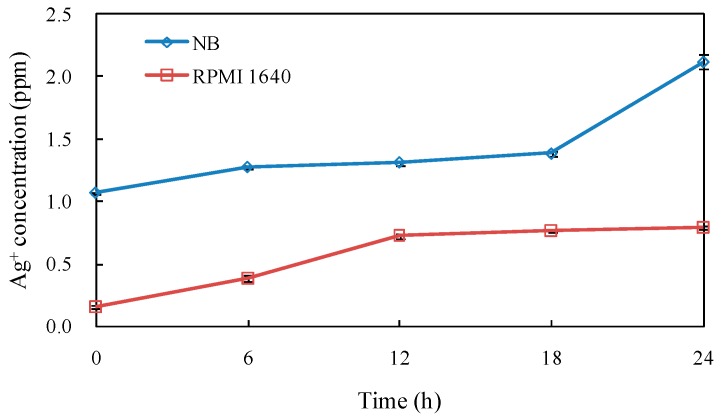
The release of Ag^+^ from AgNPs in NB and RPMI 1640 medium.

The content of Ag^+^ in nutrient broth (NB) and RPMI 1640 medium was detected in 24 h (Figure 4). The results showed that AgNPs could release Ag^+^ slowly, which reached the highest content of 2.12 and 0.79 ppm in NB and RPMI 1640 medium, respectively.

### 2.3. Antibacterial Activity of AgNPs

**Figure 5:**
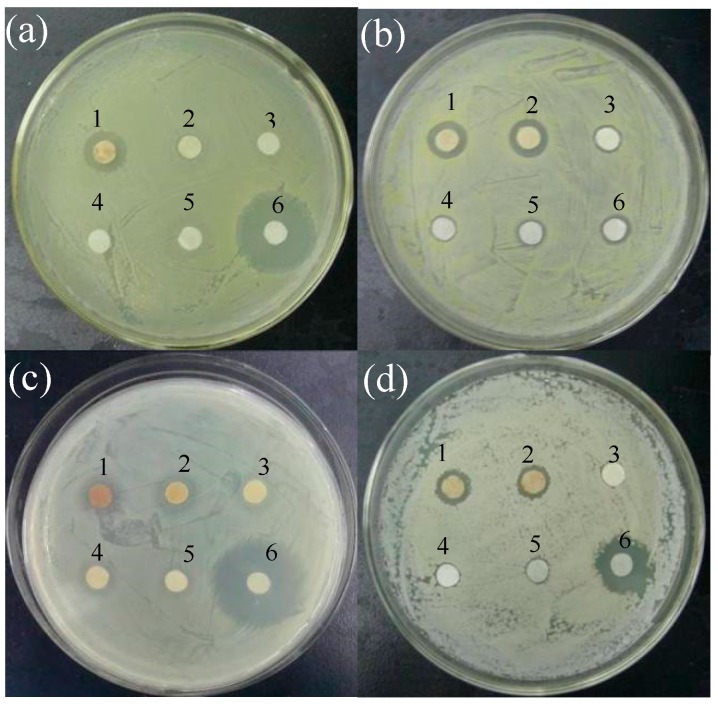
Activity of AgNPs against human pathogen: (**a**) *E. coli*; (**b**) *S. aureus*; (**c**) *S. paratyphi* B; and (**d**) *B. subtilis*, depicting zones of inhibition of: 128 μg/mL (1); 32 μg/mL (2); 8 μg/mL (3); 2 μg/mL (4); 1699 μg/mL AgNO_3_ control (5); and 1 mM gentamycin sulfate (6).

**Figure 6:**
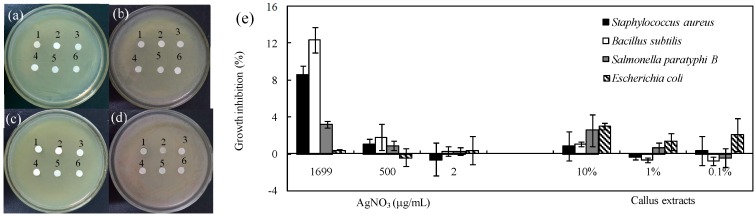
Activity (**a**–**d**) and growth inhibition (**e**) of: AgNO_3_ at 1699 μg/mL (1); 500 μg/mL (2); and 2 μg/mL (3), and callus extracts at: 10% (4); 1% (5); and 0.1% (6), against human pathogen: (**a**) *E. coli*; (**b**) *S. aureus*; (**c**) *S. paratyphi* B; and (**d**) *B. subtilis*.

**Figure 7:**
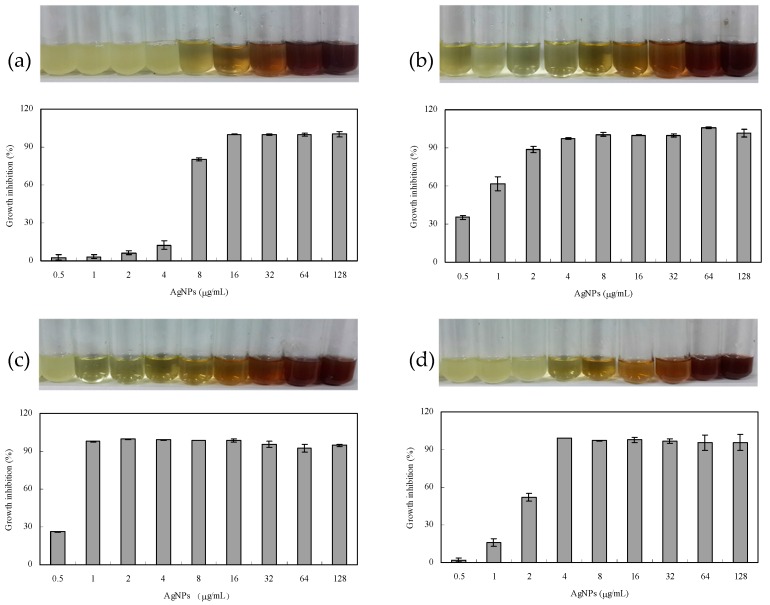
Determination of the minimum inhibitory concentration (MIC) against: *E. coli* (**a**); *S. aureus* (**b**); *B. subtilis* (**c**); and *S. paratyphi* B (**d**).

The AgNPs had concentration-dependent inhibitory effect on all testing human pathogenic bacteria (Figure 5). The growth of *Staphylococcus* *aureus*, *Salmonella* *paratyphi* B and *Bacillus* *subtilis* was inhibited by AgNPs even at 2 μg/mL. The inhibition of AgNPs against Gram-positive bacteria (*S. aureus* and *B. subtilis*) was stronger than that of Gram-negative bacteria (*Escherichia coli* and *S. paratyphi* B). AgNO_3_ control at 1699 μg/mL (10 mM) showed no antibacterial activity against *E. coli* and lower activity against other three bacteria. The control gentamycin sulfate at 1 mM had greater inhibitory effect on gram-negative bacteria *E. coli* and *S. paratyphi* B but weaker effect on gram-positive bacteria *S. aureus* and *B. subtilis*. The control of callus extracts at different concentrations (0.1%–10%) had no inhibition effects against all tested bacteria (Figure 6). We further tested antibacterial activity of AgNPs by the minimum inhibitory concentration (MIC). As shown in Figure 7, antibacterial activity increased with the increasing concentration of AgNPs. MIC of AgNPs was 8, 1, 1 and 4 μg/mL against *E. coli*, *S. aureus*, *B. subtilis* and *S. paratyphi* B, respectively.

### 2.4. Cytotoxicity Experiments

**Figure 8:**
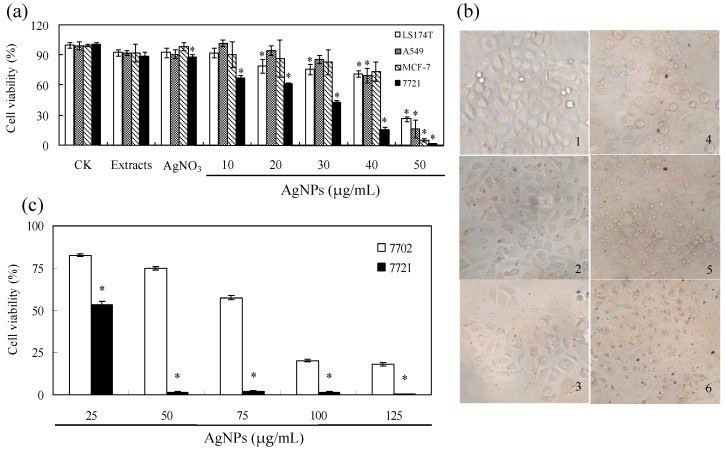
Dose-dependent cytotoxic activity of AgNPs (**a**); and the induced morphology injury of SMMC-7721 cells (**b**). (**a**) LS174T, A549, MCF-7 and SMMC-7721 cells were incubated with 0, 10, 20, 30, 40 and 50 μg/mL AgNPs, 10% (v/v) callus extracts as well as 0.79 ppm AgNO_3_. * *p* < 0.01 vs. control. HL-7702 and SMMC-7721 cells were incubated with 0, 25, 50, 75, 100 and 125 μg/mL AgNPs. * *p* < 0.01 vs. HL-7702. Data presented are the means ± SD of results from three independent experiments. (**c**) Cells were treated without (1); and with 10 (2); 20 (3); 30 (4); 40 (5); and 50 (6) μg/mL AgNPs.

**Figure 9:**
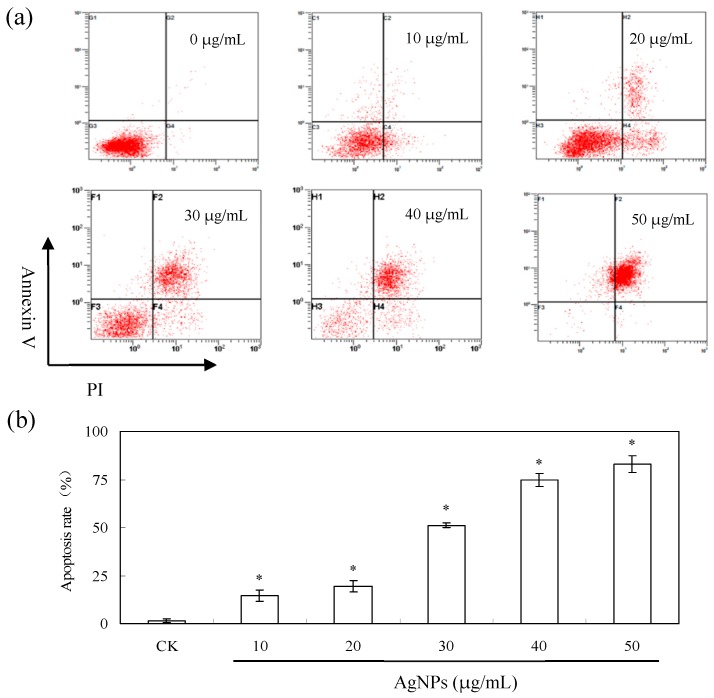
Apoptosis analysis of SMMC-7721 cells treated by AgNPs. (**a**) Flow cytometry analysis of apoptosis in SMMC-7721 cells stained with Annexin V-FITC/PI. Cells were incubated with 0, 10, 20, 30, 40 and 50 μg/mL AgNPs. (**b**) A dose-dependent increase in the percentage of apoptosis rate. * *p* < 0.01 vs. control.

As shown in Figure 8a, 10% (v/v) callus extracts had no obvious cytotoxicity while AgNO_3_ control at 0.79 ppm had no cytotoxic effect on human colon adenocarcinoma cells (LS174T), lung adenocarcinoma cells (A549) and breast cancer cells (MCF-7) but weak cytotoxic effect on human hepatoma cells (SMMC-7721) (87.8% cell viability). AgNPs could reduce all tested cancer cells viability in a dose dependent manner. SMMC-7721 cells were the most sensitive tumors cells with the IC_50_ of 27.75 μg/mL thus we chose SMMC-7721 for further research. The cellular morphology of SMMC-7721 cells were observed and photographed under microscopy (Figure 8c). The shape of cells was altered obviously when 30 μg/mL AgNPs was applied and a large number of dead cells were presented at AgNPs concentration of 40 and 50 μg/mL, which was in accord with the results of 3-(4,5-dimethylthiazol-2-yl)-2,5-diphenyltetrazolium bromide (MTT) assay (Figure 7a). Furthermore, apoptosis of SMMC-7721 cells was measured by flow cytometry (Figure 9). Apoptosis rate was increased as the concentration of AgNPs increased. On the other hand, we investigated the cytotoxicity of AgNPs against normal human liver cells HL-7702. The results indicated AgNPs inhibited the growth of HL-7702 at higher concentration (Figure 8b). IC_50_ value of AgNPs on HL-7702 cells was 81.39 μg/mL. The difference of induced cell viability between cancer cells (SMMC-7721) and normal HL-7702 cells was significant (*p* < 0.01).

## 3. Discussion

Extracts of *T. yunnanensis* have been used extensively to produce various active anticancer compounds such as taxane diterpenoids and polysaccharides [20,21,22]. In previous studies, extracts of *T. yunnanensis* and the purified compounds showed excellent cytotoxic effect against human breast adenocarcinoma cells (MCF-7), human chronic myeloblastic leukemia cells (K562) [22], human cervix carcinoma Hela cells, human fibroma carcinoma HT1080 cells [21], metastatic ovary carcinoma, and colon, head and non-small cell lung cancer [23]. The excellent anticancer effect caused its widely application in clinic. Other researchers found the extracts could be used as an anti-osteoporotic agent [24], an antiallergic reagent [25] as well as a hypoglycemic agent for diabetes [26]. In our research, we used the callus extract of *T. yunnanensis* to synthesize AgNPs. The biosynthesized AgNPs had a size range from 6.4 to 27.2 nm, which was much smaller than that of many AgNPs synthesized by plant extracts such as *Alternanthera dentate* (50–100 nm), *Acorus calamus* (31.83 nm), *Cymbopogan citrates* (32 nm), *Centella asiatica* (30–50 nm), *Psoralea corylifolia* (100–110 nm) and *Melia dubia* (35 nm) [11]. The synthesized AgNPs had significant activity of growth inhibition against human pathogenic bacteria and cancer cells with lower cytotoxicity against normal cells. Herein, the novel application of extracts of *T. yunnanensis* in green synthesis of AgNPs was reported for the first time.

Previous studies have shown that *T. yunnanensis* extracts contain secondary metabolites including taxane diterpenoids, rearranged abietanes, lignans, polysaccharides and phenolic compounds [25,27,28]. It was presumed that plant polysaccharides and phenolic compounds may act as reducing and capping agents for green synthesis of AgNPs [5]. In our experiment (Figure 3b,c), groups such as aromatic ring attached to AgNPs could not be removed by washing with water, but could be removed by alcohol. AgNPs was stable in the water even after two weeks but the stability decreased after washed by alcohol. These results indicated lignans or other secondary metabolites in the extracts might play roles in the stability of AgNPs.

Various factors including concentration of plant extracts and Ag^+^, temperature and exposure time could affect the biosynthesis of AgNPs [29,30]. Qin et al. reported that the average size of AgNPs decreased as pH of the reaction system increased from 6.0 to 10.5 [31]. In our research, the absorption peak shift towards the shorter wavelength side from pH 7 to 10, indicating the decrease of AgNPs average size [19]. However, the maximum absorption wavelength of mixture at pH 11 showed a red shift relative to that at pH 10, which may cause by the accumulation of AgNPs [32].

AgNPs promoted the usage of silver as an important antibacterial agent because of its smaller sizes, higher surface areas and greater activities [1]. Our study showed that the biosynthesized AgNPs had significant antibacterial activity against the clinical bacteria strains (*S. aureus*, *B. subtilis*, *E. coli* and *S. paratyphi* B). Sankar et al. found the antibacterial activity of AgNPs synthesized by *Origanum vulgare* against *E. coli* and *S. paratyphi* [33]. The AgNPs synthesized by *Acacia leucophloea* extract had antibacterial activity against *S. aureus* [34]. AgNPs synthesized by pine mushroom (*Tricholoma matsutake*) extracts could be a potential antimicrobial agent against *E. coli* and *B. subtilis* [35]. Kim et al. reported Gram-negative strains were more sensitive to AgNPs than Gram-positive strains because of their difference on membrane structure [36]. However, Ruparelia et al. found Gram-negative strains were more resistant to AgNPs compared to Gram-positive strains, indicating other factors might play roles besides membrane structure [37]. In our research, the inhibition of AgNPs against Gram-positive bacteria (*S. aureus* and *B. subtilis*) was stronger than that of Gram-negative bacteria (*E. coli* and *S. paratyphi* B) as shown in Figure 5 and Figure 6, which was in accordance with Ruparelia’s results [37]. In our study, AgNO_3_ control showed little antibacterial activity against the tested bacteria and the concentration of AgNO_3_ control (1699 μg/mL, equal to 10 mM) was much higher than that of Ag^+^ released in NB medium (Figure 4). Furthermore, NB medium with high concentration of sodium chloride (5.0 g/L) could interfere with the action of released Ag^+^ by precipitating of Cl^−^. On the other hand, callus extract had no growth inhibition effects against the tested bacteria (Figure 6). Those results suggested that the antibacterial activity of AgNPs in our experiments was not related to the release of Ag^+^ ions or callus extracts. It has been assumed that the released Ag^+^ could reacted with sulfur-containing amino acids inside or outside the cell membrane, phosphorus moieties in DNA, while small sized AgNPs could create pores on bacterial wall, thereby affecting bacterial cell viability [38]. The AgNPs of less than 10 nm diameters attached to cell wall caused perforation of the cell membrane of green fluorescent protein (GFP)-expressing recombinant *E. coli*, which lead to the cell death [39]. It remains to be further explored whether our fabricated AgNPs can penetrate and disrupt bacterial membranes.

Our study demonstrated that the biosynthesized AgNPs had significant dose-dependent growth inhibition in four human cancer cells. The IC_50_ of AgNPs was 40.3 μg/mL for A549 and 42.2 μg/mL for MCF-7, suggesting a moderate cytotoxic effect on both cell lines. It has been reported that the IC_50_ value against A549 cells was 100, 40 and 30 μg/mL, respectively, for different AgNPs synthesized by extracts of *Origanum* *vulgare, Gossypium hirsutum* and *Cleistanthus collinus* [33,40,41] and IC_50_ values of 17.41, 50 and 7.19 μg/mL for MCF-7 cells were presented by the fabricated AgNPs with bacteria extracts, *Annona squamosa* leaf and *Cassia fistula* flowers, respectively [42,43,44]. We also tested the cytotoxic effect of AgNPs against human colon adenocarcinoma cells (LS174T) and human hepatoma cells (SMMC-7721). The AgNPs exhibited excellent cytotoxic effect on SMMC-7721 cells (IC_50_ = 27.75 μg/mL) while Ag^+^ control had no cytotoxicity in LS174T, A549 and MCF-7 but weak cytotoxic effect on SMMC-7721. Moreover, the control of callus extracts also showed no cytotoxic effect on all tested cells. Previous studies revealed both nanoparticles and dissolved Ag^+^ can induce cytotoxicity synergistically [45]. Interestingly, AgNPs themselves can produce reactive oxygen species (ROS) and oxidative stress as well as the process to release Ag^+^ [46]. AgNPs could reduce adenosine triphosphate content of the cell, caused mitochondria damage and increased ROS production in a dose-dependent manner [47]. The apoptosis of SMMC-7721 cells induced by cerium oxide nanoparticles was proved to be associated with oxidative stress and the activation of mitogen-activated protein kinase (MAPK) signaling pathways [48]. The apoptotic effects of AgNPs synthesized by mycelia extracts were confirmed by activation of caspase 3 and DNA nuclear fragmentation [49]. In the present study, our fabricated AgNPs have exhibited significant cytotoxic effects. Therefore, further studies are needed in order to analyze the probable mechanism of induced apoptosis and the effects of AgNPs on mammalian immune system. Although cytotoxicity itself can be useful for cancer therapies, nonspecific oxidative damage is one of the greatest concerns for the application of AgNPs. In our present study, the higher IC_50_ value (81.39 μg/mL) of AgNPs to human liver cells (HL-7702) suggested our AgNPs were more cytotoxic to cancer cells than to normal cells in vitro. Notably, Faedmaleki et al. showed that AgNPs had a 44-time stronger inhibitory effect on HepG2 cells compared with normal cells [50]. We believe further cellular and molecular investigations on the different responses to AgNP exposure between cancer cells and normal cells might be helpful for a better understanding of unique mechanisms of cytotoxic actions of AgNPs. Although AgNPs as an antitumor agent can provide new opportunity for medical science, more studies are still needed to advance to clinical translation. It has been reported that AgNP surface could be functionalized with hydrophilic molecules such as polyethylene glycol (PEG) to avoid the removal by macrophage [51]. Braun et al developed etching technique to enable AgNPs to target tumor cells, and rapidly disassemble and remove AgNPs outside living cells, which minimized the off-target toxicity [52]. The treatment of targeted human ovary cancer cells by AgNPs conjugated with folic acid was conducted through site specificity by irradiating cells with a continuous wave near-infrared laser [53]. Zhao et al. developed successfully a multifunctional magnetic Fe_3_O_4_/AgNPs with a monoclonal antibody C225 targeting to the epidermal growth factor receptor (EGFR), an attractive target of many cancers [54]. Considering the biocompatible nature of the green synthesized AgNPs and potent cytotoxicity towards cancer cells, we expect that our fabricated AgNPs could be conjugated peripherally with targeting moieties. The AgNPs may be conjugated to both a chemotherapeutic agent such as docetaxel or methotrexate, and a targeting ligand such as folic acid to certain breast cancer and ovarian cancer cells of folate overexpression [55] or chlorotaxin with a high affinity for tumors of neuroectodermal origin, through a poly(ethylene glycol) linker [56]. Combining monoclonal antibodies such as EGFR-specific antibody C225 with the fabricated AgNPs is also a good strategy to improved efficacy and reduce toxicity on neighboring healthy tissues.

## 4. Materials and Methods

### 4.1. Preparation of Callus Extracts of T. yunnanensis

Callus cultures of *T. yunnanensis* were induced from young stems and subcultured on solid MS medium supplemented with 1.5 mg/L 2,4-dichlorophenoxy acetic acid and 0.5 mg/L kinetin. Callus were cultured for 4 weeks and then harvested. Callus extracts were prepared by grinding 50 g fresh callus in 250 mL sterile distilled water, boiled for 5 min and filtered through Whatman No. 1 filter paper [7]. The extracts were stored at 4 °C and used in 1 week.

### 4.2. Synthesis of AgNPs

Callus extracts (1 mL) were added to 9 mL of 1 mM aqueous AgNO_3_ solution. The reaction mixture was stirred properly and incubated for different time (10, 20, 30, 60, 90 and 120 min) and at different pH (7, 8, 9, 10 and 11) to optimize the reaction condition. Reduction of silver ions was monitored by measuring the absorbance of the reaction mixture from 300 to 600 nm using UV-vis spectrophotometer (UV-2600, Shimadzu Corporation, Kyoto, Japan) to find the absorbance peak [10]. The mixture solution was centrifuged at 22,000 *g* for 30 min [57] and the produced pellets were washed three times with distilled water and dried in a freeze drier to remove excess silver ions.

### 4.3. Characterization of AgNPs

The size and morphology of the synthesized nanoparticles were recorded by TEM (H-600, Hitachi, Osaka, Japan). Zetasizer Nano (ZEN3600, Malvern, Worchestershire, UK) was used to measure the zetapotential of the AgNPs. XRD measurement was carried out using Cu-Kα radiation source in power diffractometer (X’Pert Pro MPD, Philips, Eindhoven, The Netherlands). To obtain information about chemical groups present around the AgNPs for their stabilization, the freeze-dried powder of callus extracts, water-washed AgNPs and alcohol-washed AgNPs were ground with KBr pellets and subjected to FTIR analysis (Model FTS 7000, Varian Inc., Palo Alto, CA, USA). The AgNPs washed by NB medium (peptone, 5.0 g/L; Yeast extract, 2.0 g/L; meat extract, 1.0 g/L; sodium chloride, 5.0 g/L; pH 7.0; Sigma-Aldrich, St. Louis, MO, USA) as well as RPMI1640 medium (Gibco, Gaithersburg, MD, USA) were also analyzed by FTIR.

AgNPs were dissolved with NB and RPMI 1640 medium at 100 μg/mL. The samples were taken at different time, centrifuged and digested with 5% nitrate acid for 24 h. The release of Ag^+^ from AgNPs in the medium was determined by atomic absorption spectroscopy (SpetrAA 240, Varian Inc., Palo Alto, CA, USA).

### 4.4. Antibacterial Activity

The antibacterial activity of AgNPs against pathogenic microorganisms was examined using the standard disc diffusion method [58]. Bacterial strains *E. coli* CMCC(B)44102, *S. aureus* CMCC(B)26003, *B. subtilis* CMCC(B)63501 and *S. paratyphi* BCMCC(B)50094 were purchased from China General Microbiological Culture Collection Center (CGMCC) and cultured in NB media at 37 °C. The overnight grown bacterial suspensions were swabbed on NB plates. Whatman filter paper (No. 1) discs of 6 mm diameter were impregnated with 20 μL of 2, 8, 32 and 128 μg/mL solution of AgNPs in sterile water. AgNO_3_ at 1699 μg/mL and gentamycin sulfate at 1 mM were used as control. The discs were evaporated and then impregnated on the plates. The plates containing bacteria and discs were held at 37 °C for 24 h then inhibition zones were observed to evaluate antibacterial activity of AgNPs preliminary.

The growth inhibition was further determined by turbidimetric study. Bacterial was suspended in NB media and the bacterial suspension (1 mL, 10^6^ colony-forming units/mL) were mixed with 1 mL solution of AgNPs with the concentration of 0, 1, 2, 4, 8, 16, 32, 64, 128 and 256 μg/mL. The mixture was cultured at 37 °C for 24 h then the optical density at 600 nm was measured by a spectrophotometer (PC22, Lengguang Tech., Shanghai, China). The antibacterial activity of AgNPs was tested by determining its MIC [59].

### 4.5. Cytotoxicity Experiments

#### 4.5.1. Cell Culture

Human colon adenocarcinoma cells (LS174T), human lung adenocarcinoma cells (A549), human breast cancer cells (MCF-7), human hepatoma cells (SMMC-7721) and human liver cells (HL-7702) were grown in RPMI 1640 medium in a 37 °C, 5% CO_2_ environment. The medium was added 10% (v/v) fetal bovine serum (Tianhang Biotechnology Company, Zhejiang, China), 100 U/mL penicillin and 100 μg/mL streptomycin (Beyotime Biotechnology, Haimen, China).

#### 4.5.2. Cell Viability Assay

The cell viability was measured by the modified (MTT) method [60]. Cells were collected, centrifuged and adjusted the density to 1.5 × 10^5^/mL with the medium. Cells in 100 μL medium were seeded per well in 96-well plates and exposed with AgNPs of different concentrations for 24 h after adhering to the plates. Ten-percent (v/v) callus extracts and 0.79 ppm AgNO_3_ were used as control. Then, 10 μL MTT (5 mg/mL in phosphate buffer saline PBS) was added to each well. The cells were then incubated for another 4 h in a 37 °C, 5% CO_2_ environment and then 10% sodium dodecyl sulfate was added to each well. The 570 nm absorbance was detected with theKLx808microplate reader (Bio-Tek, Winooski, VT, USA). The experiment was repeated three independent times and the cells survival rate was calculated. Data are the mean ± standard deviation (SD) and a student’s t-test was used for statistical comparisons of two means. Differences in means were considered to be significant for *p* values < 0.01.

#### 4.5.3. Flow Cytometry Analysis of Apoptosis

The number of apoptotic cell induced by AgNPs with different concentrations was measured by flow cytometry using Annexin V-FITC-PI kit. TheAnnexin V-PI assay evaluates phosphatidylserine translocation from the inner to the outer layer of the plasma membrane which is an event typically associated with apoptosis. SMMC-7721 cells (2 × 10^5^ per well) were seeded into 6-well plates and treated with 0, 10, 20, 30, 40 and 50 μg/mL AgNPs. After treatment with AgNPs for 24 h, cells were harvested, rinsed twice with ice-PBS and resuspended in 200 μL binding buffer. Two microliters Annexin V-FITC and 2 μL PI were added and then the mixture was incubated at room temperature for 10 min in the dark. Finally, the cells were analyzed by flow cytometry (FC500, Beckman-Coulter, Hialeah, FL, USA) [48].

## 5. Conclusions

In conclusion, the biosynthesis of AgNPs by extracts from *T. yunnanensis* callus was described. The biosynthesized AgNPs had spherical shapes with particle size ranging from 6.4 to 27.2 nm. XRD pattern confirmed the presence of AgNPs and FTIR spectra indicated phytochemicals in the extracts may be involved in the synthesis and stability of AgNPs. Antibacterial assays using AgNPs revealed creditable activity against *E. coli*, *S. aureus*, *S. paratyphi* B and *B. subtilis* while cytotoxicity assays exhibited excellent activity of AgNPs against LS174T, A549, MCF-7 and SMMC-7721 cancer cells but lower activity against normal human liver cells. Our research explored a new application of *T. yunnanensis* callus and suggested that the AgNPs synthesized by environment-friendly method could be used as antibacterial and anticancer agents.

## Supplementary Materials

The following are available online at www.mdpi.com/2079-4991/6/9/160/s1.

## Abbreviations

The following abbreviations are used in this manuscript:

AgNPs	Silver nanoparticles
FTIR	Fourier transform infrared spectroscopy
MIC	Minimum inhibitory concentration
MTT	3-(4,5-Dimethylthiazol-2-yl)-2,5-diphenyltetrazolium bromide
PBS	Phosphate buffered saline
ROS	Reactive oxygen species
TEM	Transmission electron microscopy
XRD	X-ray diffraction
