# Supplementary material for: Biosynthesis of Silver Nanoparticles Using Taxus yunnanensis Callus and Their Antibacterial Activity and Cytotoxicity in Human Cancer Cells
Source: Nanomaterials (Basel). 2016 Sep 1;6(9):160. doi: 10.3390/nano6090160 (PMC5224640; doi:10.3390/nano6090160)
Supplement: Supplementary file 1 [file nanomaterials-06-00160-s001.pdf]

Supplementary Materials: Biosynthesis of Silver Nanoparticles Using *Taxus yunnanensis* Callus and Their Antibacterial Activity and Cytotoxicity in Human Cancer Cells

Qian Hua Xia, Yan Jun Ma and Jian Wen Wang

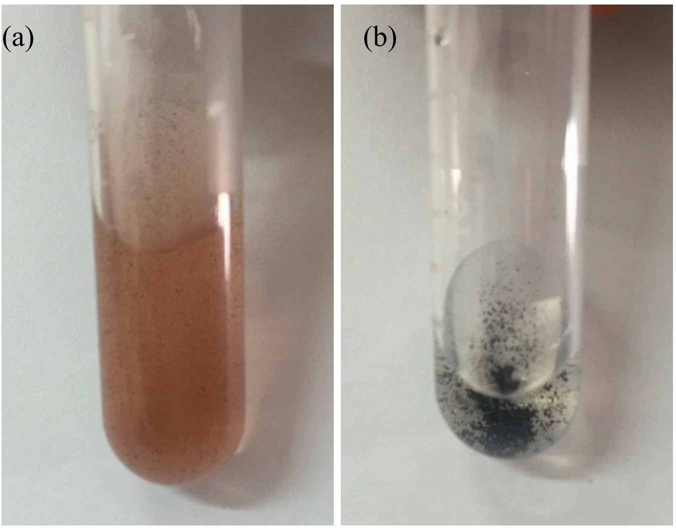

Figure S1. AgNPs washed by alcohol (**a**) and the dry powder of AgNPs redispersed into water after alcohol washing (**b**).
